# Influenza Virus Surveillance in Coordinated Swine Production Systems, United States

**DOI:** 10.3201/eid2110.140633

**Published:** 2015-10

**Authors:** Bryan S. Kaplan, Jennifer DeBeauchamp, Evelyn Stigger-Rosser, John Franks, Jeri Carol Crumpton, Jasmine Turner, Daniel Darnell, Trushar Jeevan, Ghazi Kayali, Abbey Harding, Richard J. Webby, James F. Lowe

**Affiliations:** St. Jude Children’s Research Hospital, Memphis, Tennessee, USA (B.S. Kaplan, J. DeBeauchamp, E. Stigger-Rosser, J. Franks, J.C. Crumpton, J. Turner, D. Darnell, T. Jeevan, G. Kayali, R.J. Webby);; Lowe Consulting Ltd., Albers, Illinois, USA (A. Harding, J.F. Lowe); University of Illinois College of Veterinary Medicine, Urbana, Illinois, USA (J.F. Lowe).

**Keywords:** influenza, viruses, swine, surveillance, H1N1, H3N2, H3N2, zoonoses, United States, North America

## Abstract

To clarify the epidemiology of influenza A viruses in coordinated swine production systems to which no animals from outside the system are introduced, we conducted virologic surveillance during September 2012–September 2013. Animal age, geographic location, and farm type were found to affect the prevalence of these viruses.

Influenza A viruses (IAVs) are the etiologic agents of acute respiratory disease in many mammalian species. Although originating in wild aquatic birds, IAVs have been successful in crossing the species barrier, and specific subtypes have become endemic among humans and domestic swine populations (*1*). In the United States, influenza was first described in swine herds during the 1918 pandemic and has circulated among domestic pigs for nearly a century (*2*). The ability of swine IAVs to infect humans and cause pandemics such as that of the influenza A(H1N1) virus observed during 2009 (*3*,*4*) and the sporadic transmission of various swine influenza viruses, including H1N1 (*5*), H3N2 (*6*), and variant H3N2 (*7*), are public health concerns and highlight the need for increased vigilance and understanding of IAV epidemiology among swine.

Here we report the results from 13-months of active surveillance of IAV in coordinated swine production systems in the United States. The objectives of this study were 1) to determine the prevalence of IAV within farms in a closed production system and 2) to determine which sampled population is most affected by IAV. 

## The Study

**Figure 1:**
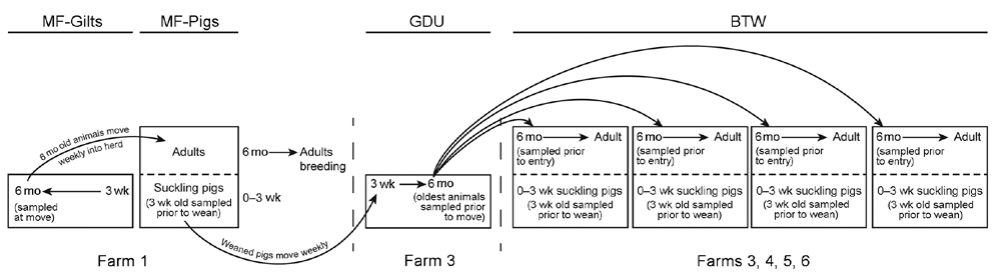
Flow of animals through a closed swine production system. Coordinated swine production systems maximize desired animal traits and weight gain. High-quality breeding sows grown and bred in multiplier farms (MF-Gilts) were sampled transfer to commercial gilt development farms (GDU), where they were sampled again at exit (6 months). At ≈3 weeks of age, piglets were sampled before weaning (MF-Pigs). Mature gilts were transported from the GDU to 1 of 4 commercial breed-to-wean (BTW) farms, where samples were collected from gilts before entry and piglets before weaning.

Multisite coordinated production systems are the common method of swine production in the United States. These systems consist of multiple farms operating in tandem, with each farm responsible for 1 stage of the production process (Figure 1). These systems are closed, meaning there are no introductions of animals from outside the system. Each farm in the system has a specific purpose: 1) to breed, gestate, farrow, and raise to the point of weaning replacement breeding stock of a specified genotype (multiplier farms [MF]); 2) to raise replacement female pigs, commonly called gilts, to 5–6 months of age for breeding (gilt development farms or units [GDU]); and 3) to breed, gestate, deliver, and raise to the point of weaning piglets specifically for meat production (breed-to-wean farms [BTW]). Gilts from GDUs are moved to MF or BTW farms for breeding. All farms house multiple age cohorts, although in different rooms or buildings. During suckling of piglets on MF and BTW farms, as well as in GDU farms, 1 cohort of piglets differing in age by <1 week are housed in a single room/building, and 1 cohort is removed before the entry of the next.

Four coordinated, multisite production systems, each consisting of 1 MF farm that includes both gilts and pigs to produce replacement female stock, 1 GDU farm to raise replacement female gilts from 3 to ≈26 weeks of age, and 4 BTW farms to raise pigs for meat, were selected to monitor the dynamics of IAV transmission in swine breeding herds. Systems are located across the United States. System 1’s MF and GDU sites are located in Illinois and its BTW sites in Georgia. Systems 2, 3, and 4 are located entirely in Illinois, Oklahoma, and Nebraska, respectively.

Nasal swab samples were collected from pigs monthly during September 2012–September 2013 from each farm in all 4 systems. The animal type and time of sampling differed on the different farm types. On the MF and BTW farms, samples were collected from 30 piglets at ≈3 weeks of age and from 30 gilts. On the MFs, 30 gilts were sampled before entry to the farm. On the GDU farms, 30 animals at ≈26 weeks of age were sampled before movement to a BTW. In the BTWs, gilts were sampled before breeding (4–8 weeks after arrival) and piglets were sampled immediately before weaning (Figure 1). This strategy enabled assessment of IAV status before the movement of animals to the next stage of production. Laboratory methods are summarized in the Technical Appendix. 

During the 13-month period spanning September 2012–September 2013, a total of 14,954 swab samples were collected and tested for the presence of the IAV matrix gene by real-time reverse transcription PCR. Of the samples collected, 741 (5.0%) tested positive, which is consistent with previous surveillance studies (*8*). 

Bivariate analysis found statistically significant correlations between infection and location in Illinois, GDU farm type, and system 2 (p<0.001 for all), but not for age (Table 1). We then constructed a logistic regression model that assessed the effect of age, system, location, and farm type on having a positive influenza result. Age was statistically significant by this model (p = 0.004); the odds ratio for piglets at weaning whose samples tested positive for IAV was 1.3 (95% CI 1.1–1.6) compared to that for gilts (Table 2), which is consistent with previous studies (*9*). However, this finding could be related to interaction between age and state/system. System 2 again had higher odds of positive results (OR 1.7, 95% CI 1.3–2.3) compared with system 1. MF pigs were found to have a lower risk for infection (OR 0.7, 95% CI 0.5–0.9) and GDU pigs to have a higher risk for infection (OR 1.6, 95% CI 1.2–2.1) when compared with BTW pigs. Finally, Illinois had higher odds for IAV infection (OR 3.2, 95% CI 2.6–4.0) compared with the other 3 states.

**Table 1:** Epidemiologic data for influenza A virus among swine in coordinated swine production systems, United States, September 2012–September 2013*

Variable	No. samples	No. (%) positive samples	p value
State*			
Georgia	2,520	73 (2.9)	
Illinois	4,490	408 (9.1)	<0.001
Nebraska	3,894	126 (3.2)	
Oklahoma	4,050	134 (3.3)	
Age group			
Gilt	8,028	375 (4.7)	0.85
Piglet	6,926	366 (5.3)	
Farm type			
MF, gilts†	1,526	67 (4.4)	
MF, piglets	1,559	72 (4.6)	
GDU	1,455	115 (7.9)	<0.001
BTW	10,414	487 (4.7)	
System			
1	3,673	142 (3.9)	
2	3,337	339 (10.2)	<0.001
3	4,050	134 (3.3)	
4	3,894	126 (3.2)	

*Piglets were sampled before weaning; gilts were sampled at entry to MFs and before moving from GDUs to BTWs. MF, multiplier farm; GDU, gilt development unit; BTW, breed-to-wean.

**Table 2:** Correlation between age, state, farm type or production system, and influenza A virus status in swine in coordinated production systems, United States, September 2012–September 2013*

Factor	aOR (95% CI)	p value
Age group		
Piglet vs. gilt	1.3 (1.1–1.6)	0.004
State		
Illinois vs. Oklahoma	1.9 (1.4–2.6)	<0.001
Farm type		
MF piglets vs. BTW	0.7 (0.5–0.9)	0.011
GDU vs. BTW	1.6 (1.2–2.1)	<0.001
System number		
2 vs. 1	1.7 (1.3–2.3)	<0.001

*Only statistically significant factors are shown; full analysis is provided in the Technical Appendix. aOR, adjusted odds ratio; CI, confidence interval; MF, multiplier farm; GDU, gilt development unit; BTW, breed-to-wean.

**Figure 2:**
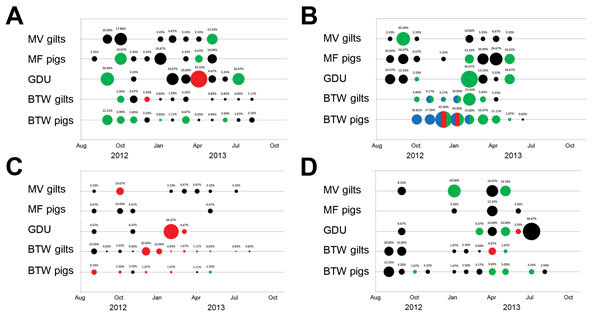
Prevalence of influenza A virus by system, farm type, and month, United States, September 2012–September 2013, positive RNA nasal swab samples were categorized by age range (gilt, female pig 5–6 months of age or piglet, ≈3 weeks of age) and production point farm (multiplier farms [MF] Gilt, MF Pig, GDU, BTW Gilt, BTW Pig). Each circle depicts a month during which positive influenza samples were collected; percentage of positive swabs is listed above each circle. Influenza A subtypes are indicated by circle color: green, H1N1; blue, H1N2; red, H3N2; black, untyped. Multicolored circles indicate the detection of >1 subtype.

IAV subtypes were determined for 25.2% of the IAV positive samples (Figure 2). All 3 common porcine influenza subtypes (H1N1, H1N2, and H3N2) were detected during the 13-month surveillance period. Increased prevalence of IAV was detected in piglets (MF pigs, GDU, BTW pigs) in all 4 systems, particularly in systems 1, 2, and 4, from winter to early summer (Figure 2, panels A, B, D), which is consistent with other studies (*8*). Multiple subtype detection occurred only on BTW farms in system 2 (Figure 2, panel B).

## Conclusions

We found that IAV infection was present at all stages of swine production within coordinated production systems. Animal age, geographic location, and type of farm affected risk for infection. We also found continuing virus circulation in all populations year round, although prevalence was higher from winter through early summer. To fully elucidate the factors that contribute to persistent IAV infection in swine farms and therefore develop evidence-based control strategies, further research is needed. 

Technical Appendix Logistic regression model of influenza A virus status among swine in coordinated production systems, USA, September 2012-September 2013.

## References

[1] Webster RG, Bean WJ, Gorman OT, Chambers TM, Kawaoka Y. Evolution and ecology of influenza A viruses. Microbiol Rev. 1992;56:152–79 . 1579108 10.1128/mr.56.1.152-179.1992 PMC372859

[2] Brown IH. The epidemiology and evolution of influenza viruses in pigs. Vet Microbiol. 2000;74:29–46 . 10.1016/S0378-1135(00)00164-4 10799776

[3] Garten RJ, Davis CT, Russell CA, Shu B, Lindstrom S, Balish A, Antigenic and genetic characteristics of swine-origin 2009 A(H1N1) influenza viruses circulating in humans. Science. 2009;325:197–201. 10.1126/science.1176225 19465683 PMC3250984

[4] Dawood FS, Jain S, Finelli L, Shaw MW, Lindstrom S, Garten RJ, Novel Swine-Origin Influenza A (H1N1) Virus Investigation Team, Emergence of a novel swine-origin influenza A (H1N1) virus in humans. N Engl J Med. 2009;360:2605–15. 10.1056/NEJMoa0903810 19423869

[5] Epperson S, Jhung M, Richards S, Quinlisk P, Ball L, Moll M, Human infections with influenza A(H3N2) variant virus in the United States, 2011–2012. Clin Infect Dis. 2013;57(Suppl 1):S4–11. 10.1093/cid/cit272 23794729

[6] Wong KK, Gambhir M, Finelli L, Swerdlow DL, Ostroff S, Reed C. Transmissibility of variant influenza from swine to humans: a modeling approach. Clin Infect Dis. 2013;57(Suppl 1):S16–22. 10.1093/cid/cit303 23794727 PMC4593498

[7] Freidl GS, Meijer A, de Bruin E, de Nardi M, Munoz O, Capua I, Influenza at the animal-human interface: a review of the literature for virological evidence of human infection with swine or avian influenza viruses other than A(H5N1). Euro Surveill. 2014;19:20793. 24832117 10.2807/1560-7917.es2014.19.18.20793

[8] Corzo CA, Culhane M, Juleen K, Stigger-Rosser E, Ducatez MF, Webby RJ, Active surveillance for influenza A virus among swine, midwestern United States, 2009–2011. Emerg Infect Dis. 2013;19:954–60. 10.3201/eid1906.121637 23735740 PMC3713829

[9] Allerson MW, Davies PR, Gramer MR, Torremorell M. Infection dynamics of pandemic 2009 H1N1 influenza virus in a two-site swine herd. Transbound Emerg Dis. 2014;61:490–9. 10.1111/tbed.12053 23294593

