# Supplementary material for: Influenza Virus Surveillance in Coordinated Swine Production Systems, United States
Source: Emerg Infect Dis. 2015 Oct;21(10):1834–6. doi: 10.3201/eid2110.140633 (PMC4593420; doi:10.3201/eid2110.140633)
Supplement: Technical Appendix — Logistic regression model of influenza A virus status among swine in coordinated production systems, USA, September 2012-September 2013. [file 14-0633-Techapp-s1.pdf]

Article DOI: http://dx.doi.org/10.3201/eid2110.140633

Surveillance of Influenza Virus A in Coordinated Swine Production Systems, United States

Technical Appendix

Materials and Methods

Sample Testing

All nasal swabs were screened for influenza A virus in the Department of Infectious Diseases, Virology Division at St. Jude Children's Research Hospital (Memphis, TN, USA). Upon receipt, nasal swabs were stored in PBS supplemented with penicillin (20,000 units/mL), streptomycin (4,000 units/mL), polymixin B (2,000 units/mL), gentamicin (0.5mg/mL) and stored at 4°C until testing. Samples were tested for presence of the IAV matrix gene by RRT-PCR using TaqMan probes, 4x Fast Virus RT-PCR kit (Life Technologies), in ABI 7500 Fast PCR. Primer and probe sequences are as follows: Forward primer: 5′-GACCRATCCTGTCACCTCTGAC-3′, Reverse Primer: 5′-AGGGCATTYTGGACAAAKCGTCTA-3′, Probe: 5′-6FAM-TGCAGTCCTCGCTCACTGGGCACG-TAMRA-3′. Samples were classified as positive with a Ct value ≤36. Positive samples underwent further testing for HA and NA subtype by using Swine Influenza Virus RT-PCR duplex assays for H1/H3 and N1/N2 (Life Technologies) with VetMAX-Plus One Step RT-PCR Kit (Ambion) according to manufacturer's instructions. This study received approval from the institutional animal care and use committee (IACUC) at St. Jude Children's Research Hospital.

Statistical Analyses

The proportion of samples that were positive according to the different study variables was determined. The Pearson χ^2 test was used. Statistical analysis was performed by using PASW Statistics 18 (SPSS) (IBM, Armonk, NY). Stepwise logistic regression was used to study the association between study variables and influenza infection. All study variables were entered in the model. To assess interaction between age and the other variables, we constructed several logistic regression models that included age and one of the other study variables with and without the interaction term.

Technical Appendix Table 1. Logistic regression model of influenza A virus status among swine in coordinated production systems, USA, September 2012-September 2013*†

Factor	*P* value
Age group	
Piglet	0.004
Gilt	Reference
State	
Illinois	<0.001
Georgia	NS
Nebraska	NS
Oklahoma	Reference
Farm Type	
MF-Pigs	0.011
GDU	<0.001
MF-Gilts	NS
BTW	Reference
System	
2	<0.001
3	NS
4	NS
1	Reference

*NS, not significant; MF, multiplier farm; GDU, gilt development unit; BTW, breed-to-wean.
β = –3.789, p-value <0.001; –2 log likelihood = 5,637.3; Nagelkerke R2 = 0.053; Hosmer and Lemeshow Test: χ^2 = 32.7, p-value<0.001.

Technical Appendix Table 2. Assessing interaction between age and other variables in studies of swine in coordinated production systems, USA, September 2012-September 2013*

Model	Variables	p-value
Age group and state		
	Age group	NS
	State (Illinois)	<0.001
Age group, state, age group†, state		
	Age group	<0.001
	State	<0.001
	Age group†State	<0.001
Age group and farm type		
	Age group	<0.001
	Farm type (GDU)	<0.001
Age group, farm type, age group†, farm type		
	Age group	<0.001
	Farm type (GDU)	<0.001
	Age†Farm type	NS
Age group and system		
	Age group	NS
	System (2)	<0.001
Age group, system, age group†, system		
	Age group	NS
	System	0.016
	Age group†, system (2)	<0.001
	Age†,system (3)	<0.001

*Interaction between age and state as well as age and system type were detected; NS, not significant; GDU, gilt development unit.
†Indicates interaction between the variables.
